# Alteration of the Mitochondrial Effects of Ceria Nanoparticles by Gold: An Approach for the Mitochondrial Modulation of Cells Based on Nanomedicine

**DOI:** 10.3390/nano10040744

**Published:** 2020-04-13

**Authors:** Patricia Gutiérrez-Carcedo, Sergio Navalón, Rafael Simó, Xavier Setoain, Carolina Aparicio-Gómez, Ibane Abasolo, Victor Manuel Victor, Hermenegildo García, José Raúl Herance

**Affiliations:** 1 Medical Molecular Imaging Research Group, Vall d’Hebron Research Institute, CIBBIM-Nanomedicine, Universitat Autònoma de Barcelona (UAB) and Biomedical Imaging Group, Biomedical Research Networking Center in Bioengineering, Biomaterials and Nanomedicine (CIBER-BBN), 08035 Barcelona, Spain; biotecpat@gmail.com (P.G.-C.); carolina.aparicio@vhir.org (C.A.-G.); 2 Diabetes and Metabolism Research Unit, Vall d’Hebron Research Institute, Department of Endocrinology, Vall d’Hebron Research Institute, UAB, Biomedical Research Center in Diabetes Network and Associated Metabolic Diseases (CIBERDEM), 08035 Barcelona, Spain; rafael.simo@vhir.org; 3 Deparment of Chemistry and Instiute of Chemical Technology (CSIC-UPV), Universitat Politècnica de València, 46022 Valencia, Spain; sernaol@doctor.upv.es; 4 Hospital Clinic, Biophysics and Bioengineering Unit, Biomedicine Department, School of Medicine, University of Barcelona, and CIBER-BBN, 08036 Barcelona, Spain; setoain@clinic.cat; 5 Functional Validation & Preclinical Research (FVPR), Group of Drug Delivery & Targeting, CIBBIM-Nanomedicine, Vall d’Hebron Research Institute, UAB, CIBBER-BBN, 08035 Barcelona, Spain; ibane.abasolo@vhir.org; 6 Service of Endocrinology and Nutrition, University Hospital Doctor Peset, FISABIO, 46017 Valencia, Spain; victor.victor@uv.es; 7 CIBERehd, Department of Physiology, University of Valencia, 46010 Valencia, Spain

**Keywords:** mitochondrial function, ceria nanoparticles, gold-supported ceria nanoparticles, antioxidant, triphenylphosphonium gold-supported ceria nanoparticles

## Abstract

Ceria nanoparticles are cell compatible antioxidants whose activity can be enhanced by gold deposition and by surface functionalization with positive triphenylphosphonium units to selectively target the mitochondria. The antioxidant properties of these nanoparticles can serve as the basis of a new strategy for the treatment of several disorders exhibiting oxidative stress, such as cancer, diabetes or Alzheimer’s disease. However, all of these pathologies require a specific antioxidant according with their mechanism to remove oxidant species excess in cells and diminish their effect on mitochondrial function. The mechanism through which ceria nanoparticles neutralize oxidative stress and their effect on mitochondrial function have not been characterized yet. In the present study, the mitochondria antioxidant effect of ceria and ceria-supported gold nanoparticles, with or without triphenylphosphonium functionalization, was assessed in HeLa cells. The effect caused by ceria nanoparticles on mitochondria function in terms of mitochondrial membrane potential (∆Ψm), adenosine triphosphate (ATP) production, nuclear respiratory factor 1 (NRF1) and nuclear factor erythroid–2–like 1 (NFE2L1) was reversed by the presence of gold. Furthermore, this effect was enhanced when nanoparticles were functionalized with triphenylphosphonium. Our study illustrates how the mitochondrial antioxidant effect induced by ceria nanoparticles can be modulated by the presence of gold.

## 1. Introduction

The use of nanoparticles (NPs) in biomedicine continues to be a rapidly growing research field due to their powerful applications in medicine. Different types of NPs have been proposed for various biomedical applications such as drug delivery, bioimaging, thermotherapy and antioxidant therapy [1,2,3,4,5]. Furthermore, NPs can be functionalized to recognize specific biological targets, improving their effect; for example, triphenylphosphonium (TPP) and anti-HER2 can be used to target mitochondria [6,7] or specific tumor cells [8], respectively. Among all types of NPs proposed for biomedical applications, ceria NPs (CeO_2_) are among the most promising due to their physicochemical properties, versatility and biocompatibility [9].

CeO_2_ is a well-established antioxidant agent in biomedicine [10,11]. The antioxidant properties of CeO_2_ can be enhanced when it is combined with certain metals such as gold, silver or platinum [12,13,14,15,16], which have been proposed as biocompatible materials acting as radical scavengers and catalase-like enzymes to treat oxidative stress (OS) [14,17,18,19,20]. Furthermore, it has been suggested that CeO_2_ NPs, alone or in combination with metals, protect against radiation and have beneficial effects on different pathologies such as dementia and infection [21,22,23,24,25]. In addition, CeO_2_ can be functionalized with TPP to target CeO_2_ to the mitochondria, increasing its antioxidant behavior in cells [7].

Mitochondria are cellular organelles that play key physiological roles in cells, and are the major source of OS, as they generate a high amount of reactive oxygen species (ROS) when they do not work properly. OS is a pathological process generated by an imbalance between ROS and antioxidants, and it is associated with several disorders such as diabetes, obesity, stroke, Parkinson’s disease, Alzheimer’s disease, Friedrich’s ataxia and cancer [26,27]. Thus, OS is considered a therapeutic target to treat multiple diseases [28] since the overproduction of ROS causes oxidative damage to DNA, lipids, proteins and other biomolecules, altering their function and constituting the origin of different diseases [26,29,30,31]. The reduction of ROS overproduction in the mitochondria is orchestrated endogenously by different antioxidant systems such as the nuclear respiratory factor (NRF), composed of nuclear respiratory factor 1 (NRF1) and nuclear factor erythroid 2-related factor 1 (NFE2L1), which are related to mitochondria efficiency and the expression of endogenic antioxidant defenses, respectively [32]. The reduction of ROS can also be achieved by decreasing exposure to environmental oxidant pollutants, increasing the levels of exogenous and/or endogenous antioxidants, stabilizing the mitochondrial energy production and efficiency or using mitochondrial uncouplers [33]. Diseases with OS needed to be treated with specific antioxidants, taking into account their cellular action mechanism, mainly by determining the influence on the mitochondria function as the main ROS source. Although widespread, the use of antioxidants to prevent oxidative stress can often yield inconclusive or contradictory results due to insufficient understanding of their mode of action and their effect on mitochondrial function [16,34,35]. This is the case with several NPs that have been proposed as antioxidants in preclinical studies, for which a lack of knowledge regarding their mitochondrial action mechanism has inevitably hampered their clinical translation [36].

In this study, the effect on the mitochondrial function of CeO_2_-supported gold (AuCeO_2_) conjugated (or not) to TPP groups (TPP–AuCeO_2_) was assessed. The aim of this work is to shed light on the mitochondrial effect of these NPs, and thus, to determine their utility for therapeutic purposes.

## 2. Materials and Methods

### 2.1. Synthesis and Characterization of NPs

#### 2.1.1. Preparation of 3–Iodopropyl-Functionalized AuCeO_2_ (I–AuCeO_2_)

CeO_2_ and AuCeO_2_ were prepared following the methodology previously described by our group [20,37]. To obtain I–AuCeO_2_, AuCeO_2_ was silylated by stirring a suspension of dried AuCeO_2_ (314.5 mg) in 6 mL of an anhydrous toluene (Sigma-Aldrich, Madrid, Spain) solution containing 3–(iodopropyl)trimethoxysilane (61 mg, 0.34 mmol) (Sigma-Aldrich, Madrid, Spain) for 3 h at 85 °C. Afterwards, the mixture was allowed to cool at room temperature. Then, I–AuCeO_2_ was filtered and sequentially washed with toluene (3 × 100 mL), acetone (3 × 100 mL) and diethyl ether (2 × 100 mL) (all solvents from Sigma-Aldrich, Madrid, Spain), and later allowed to dry at room temperature under reduced pressure. The material was obtained as a brown solid, and the presence of iodopropyl groups was determined by FT-IR spectroscopy (Jasco FT-IR-460 PLUS, Easton, MD, USA), while the content of C was determined by chemical combustion analysis in a FISONS CHNOS analyzer (Fisons EA-1108-CHNS-O, Milano, Italy).

#### 2.1.2. Preparation of Triphenylphosphonium-Functionalized AuCeO_2_ (TPP–AuCeO_2_)

One hundred seventy-five milligrams of I–AuCeO_2_ were suspended in 6 mL anhydrous toluene (Sigma-Aldrich, Madrid, Spain), and then triphenylphosphine (60 mg, 0.23 mmol) (Sigma-Aldrich, Madrid, Spain) was added to this mixture. The suspension was flushed with an argon stream (1 mL/min) (Linde, Barcelona, Spain) for 10 min, and heated at reflux temperature under magnetic stirring. Then, the system was cooled to room temperature and the resulting solid was recovered by filtration and washed sequentially with toluene (3 × 100 mL), acetone (3 × 100 mL) and diethyl ether (2 × 50 mL) (all solvents from Sigma-Aldrich, Madrid, Spain). The resulting TPP–AuCeO_2_ solid was dried at room temperature under reduced pressure. TPP–AuCeO_2_ was characterized by FT-IR spectroscopy, transmission electron microscopy (TEM) (JEM-2100F, Peabody, MA, USA), dynamic light scattering (DLS) using Zetasizer Nano ZS (Malvern Instrument, Malvern, UK) and nuclear magnetic resonance spectroscopy (NMR) (Varian Gemini 3000, Palo Alto, CA, USA).

### 2.2. Cell Culture

Experiments were performed with the human cervical carcinoma cell line, HeLa cells, (ATCC^®^ CCL-2™, Manassas, VA, USA). Cells were cultured in DMEM supplemented with 10% heat-inactivated FBS, penicillin (50 U/mL) and streptomycin (50 mg/mL) (all components were from Thermo Fisher Scientific, Sant Cugat del Vallés, Spain) and incubated at 37 °C and 5% CO_2_ in a humidified atmosphere.

#### 2.2.1. Cellular Viability and Proliferation

HeLa cells were seeded on 96-well plates (Scharlab, Sentmenat, Spain) at 35,000 cells/well. After 24 h, cells were incubated in the presence of CeO_2_, AuCeO_2_ or TPP–AuCeO_2_ at 10 and 20 µg/mL for 24, 48 and 72 h. Afterwards, cellular viability and proliferation were determined following the manufacturer’s protocol using an MTT Cell Assay Kit (Merck Millipore, Madrid, Spain)). Finally, the absorbance at 590 nm was measured using a Synergy Mx plate-reader spectrophotometer (BioTek Instruments, Winooski, VT, USA). Following the manufacturer’s protocol, controls were performed using a reference cell culture media with and without cells. An additional control with NPs at 1 μg/mL in cell culture media as also used. This NP concentration was chosen considering that commonly, less than 5% of NPs present in a cell culture media become internalized in the cultured cells [38].

#### 2.2.2. Cellular Uptake and Internalization of Conjugate by Confocal Microscopy

HeLa cells were seeded at 8 × 10^5^ per dish in Nunc™ Glass culture dishes (Thermo Fisher Scientific, Sant Cugat del Vallés, Spain). After 24 h, different NPs (AuCeO_2_ or TPP–AuCeO_2_) at 20 µg/mL were added and incubated for 24 h (37 °C, 5% CO_2_ atmosphere (Linde, Barcelona, Spain)). Nuclei (Hoechst 33342 dye, 1:20,000, blue) (Life Technologies, Madrid, Spain), cell membrane (CellMaskTM, 1:10,000, green) (Life Technologies, Madrid, Spain), and mitochondria (MitoTracker™, 1:2,000, red) (Thermo Fisher Scientific, Sant Cugat del Vallés, Spain) were stained following the manufacturer’s protocol [39,40]. Fluorescence images were obtained in vivo using a FV1000-spectral confocal microscope (Olympus, Hamburg, Germany). Samples were maintained at 37 °C under 5% CO_2_ atmosphere during imaging and were illuminated simultaneously with laser light at 405 nm (exciting Hoechst, blue), 488 nm (exciting CellMask, green), and 561 nm (exciting MitoTracker, red), recording the emission from 425 to 603 nm in separate channels. The reflection signal of NPs was split by using a dichroic mirror (20/40) after irradiating at 633 nm. A Z-Stack study across the depth of the cells was performed with an interslice distance of 400 nm [41,42]. The Fiji image analysis software was used to analyze the images [43].

#### 2.2.3. Measurement of the Mitochondrial O_2_ Consumption

HeLa cells were treated with NPs for 24 h, resuspended (5 × 10^6^ cells/mL) in Hank’s Balanced Salt Solution (HBSS) (GIBCO, Thermo Fisher Scientific, Sant Cugat del Vallés, Spain) and then placed in a gas-tight chamber, and O_2_ consumption was measured with a Clark-type O_2_ electrode (Rank Brothers, (Bottisham, UK). Sodium cyanide (10^−3^ M) was used to confirm whether the O_2_ consumption was mainly mitochondrial (95–99%) or not. The O_2_ consumption rate was calculated as nmol/min/10^6^ cells and expressed as a percentage of the untreated control.

#### 2.2.4. Measurement of Mitochondrial Membrane Potential (∆Ψm) and Total ROS Production

Cells were seeded in 48-well plates, and after 24 h, they were treated with the NPs in cell culture media at a concentration of 20 µg/mL for 24 h. ∆Ψm and total ROS were assessed by fluorescence microscopy (IX81 Olympus, Hamburg, Germany) after 30 min incubation in HBSS (GIBCO, Thermo Fisher Scientific, Sant Cugat del Vallés, Spain) with fluorescent probes 5 × 10^−6^ mol/L tetramethylrhodamine methyl ester (TMRM) and 2’7’–dichlorodihydrofluorescein diacetate (DCFH–DA), and Hoechst 33342 (all probes from Sigma-Aldrich, Madrid, Spain). Cells were washed twice with HBSS and a total of 16–25 images per well were recorded with the fluorescence microscope. The fluorescence signal in individual cells was recorded with a fluorescence microscope coupled with static cytometry software (“ScanR” version 2.03.2, IX81 Olympus). Controls were performed using HBSS, cells in HBSS and NPs solved in HBSS at 1 μg/mL.

#### 2.2.5. ATP Determination

HeLa cells were grown in 96-well black plates and cultured for 48 h (37 °C, 5% CO_2_ atmosphere). Then, the cells were conditioned for 24 h in DMEM media (GIBCO, Thermo Fisher Scientific, Sant Cugat del Vallés, Spain) before treating them with the NPs (20 µg/mL). ATP measurements in cells treated and untreated with NPs were determined in triplicate by bioluminescence methodology following the manufacturer’s protocol (ATP determination Kit, ThermoFisher Scientific, Sant Cugat del Valles, Spain ) using a luminometer Synergy Mx plate reader fluorometer (BioTek Instruments, Winooski, VT, USA). Briefly, cells were washed with HBSS and 60 µL of assay buffer were added to each well. Then, 15 µL of luciferase buffer (8 ng/mL of luciferase) and 25 µL of D–luciferin buffer (2 mM of D–luciferin) was added to each well with an automatic injector, and the luminescence of each well was read in a luminometer before and after D-luciferin addition. Once the background luminescence was subtracted from each well, ATP measurements in cells treated with NPs were referred to those of the nontreated control cells. ATP assays were performed within the U20/FVPR of ICTS Nanbiosis.

#### 2.2.6. Real-Time PCR

Total RNA was extracted from cell pools after treatment with the NPs using the RNA isolation kit, following the manufacturer’s protocol (Sigma-Aldrich, Madrid, Spain). The concentration and purity of the RNA were assessed by measuring the 260/280 ratio and 260/ 230 ratio using NanoDrop™ 2000 (Thermo Fisher Scientific, Waltham, MA, USA). Briefly, 1 μg of RNA was used to synthesize cDNA with the help of a kit (Thermo Scientific, Rockford, IL, USA) and the cDNA obtained was employed for RT-PCR analysis (7500 Fast RT-PCR system, Life technologies, Camarillo, CA, USA). Expression of NRF1 and NFE2L1 was assessed using SYBR Select Master Mix (Applied Biosystems, Beverly, MA, USA). GAPDH gene (Sigma-Aldrich, Madrid, Spain) expression was used as the endogenous control. The following primer (Sigma-Aldrich, Madrid, Spain) sequences were employed:

NRF1, 5’-CGGGACAGAGTCACCATTTGA-3’ and 3’-GGGGCACTGTACAGGATTTCA-5’ NFE2L1, 5’-CGGGACAGAGTCACCATTTGA-3’ and 3’-GGGGCACTGTACAGGATTTCA-5´ GAPDH, 5’-CGCATCTTCTTTTGCGTCG-3’ and 3’-TTGAGGTCAATGAAGGGGTCA-5’. Relative quantification was performed according to the comparative 2^−ΔΔCt^ method.

#### 2.2.7. Western Blot

Total protein extracts from HeLa cells were obtained after lysis with cold RIPA buffer containing proteases inhibitor cocktail (cOmplete™, Mini, EDTA-free Protease Inhibitor Cocktail, (Merck Madrid, Spain). Protein concentration was quantified using a BCA protein assay kit (Thermo Fisher Scientific, Sant Cugat del Vallés, Spain) [44]. 40 µg of protein was used in SDS-PAGE and then transferred to PVDF membranes and incubated with primary antibodies. NRF1 and NFE2L1 antibodies (both polyclonal with human reactivity and rabbit as biological source; Sigma-Aldrich, Madrid, Spain) were incubated overnight at 4 °C with a dilution 1/1000, followed by the secondary anti-Rabbit antibody for 1 h at room temperature with a dilution 1/25000. β-Actin (monoclonal with human reactivity and mouse as biological source Sigma-Aldrich, Madrid, Spain) was used as the housekeeping protein. Finally, detection was performed with ECL (GE Healthcare Life science, Sheffield, UK) using ImageQuant LAS 4000 (GE Healthcare, Uppsala, Sweden). Signals were quantified using ImageJ software (version 1.51u, Bethesda, MD, USA).

### 2.3. Statistical Analysis

The data obtained in this study are presented as the mean ± standard error of the mean (SEM). Statistical analysis was performed using Prism 6 statistical software (GraphPad Software, Version 6.01, Prism, San Diego, CA, USA). One-way analysis of variance (ANOVA) followed by Tukey-Kramer test to evaluate the significant differences between groups were performed. Values of *p*-value of < 0.05 are indicated as * and < 0.01 as **.

## 3. Results

### 3.1. Synthesis and Characterization of Nanoparticles

**Scheme 1:**
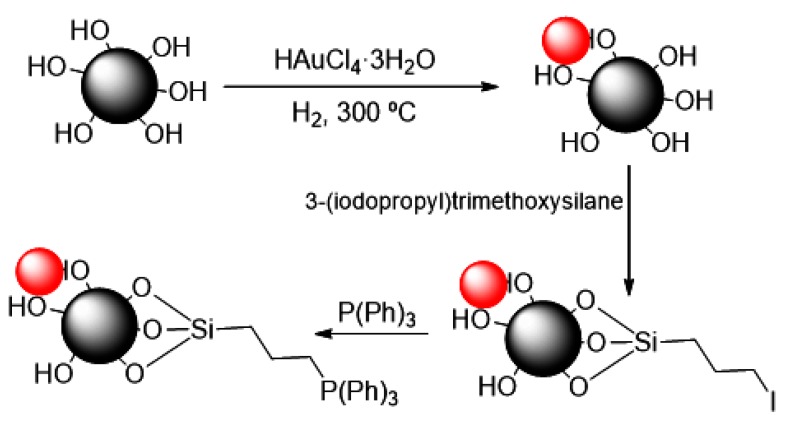
Synthetic route to obtain AuCeO_2_ and TPP–AuCeO_2_ from CeO_2_ (dark sphere). Au and CeO_2_ NPs are represented as red and grey spheres, respectively.

**Figure 1:**
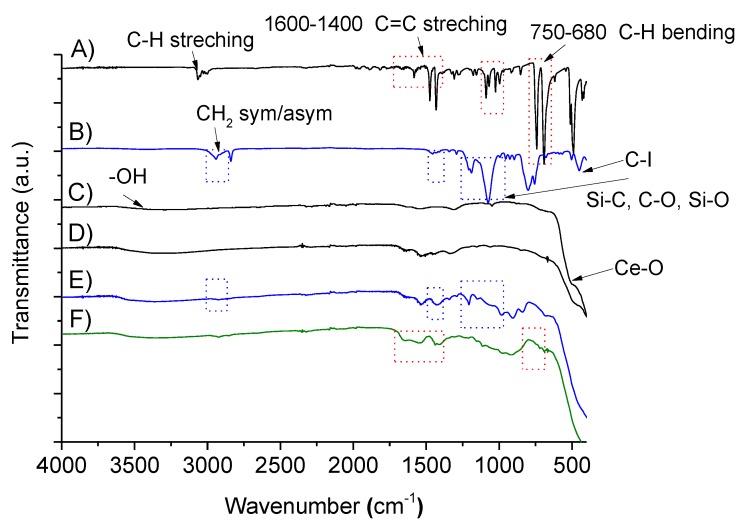
FT-IR spectra of some reagents employed to obtain TPP–AuCeO_2._ (**A**) PPh_3_, (**B**) Si(MeO)_3_–propyl–I, (**C**) CeO_2_, (**D**) AuCeO_2_, (**E**) I–AuCeO_2_, and (**F**) TPP–AuCeO_2_. The main bands employed to identify different functional groups are labelled in the spectra.

**Figure 2:**
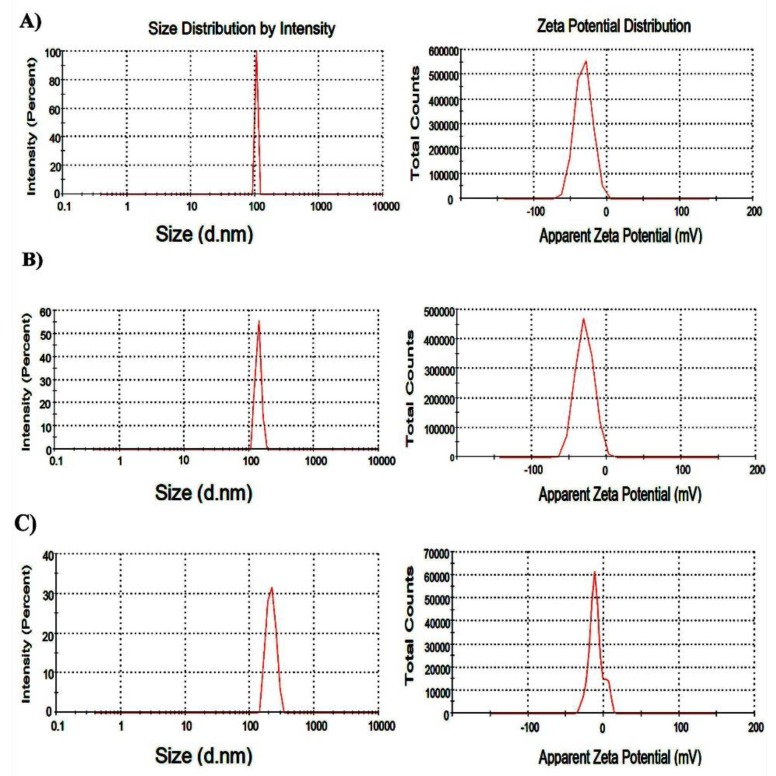
Dynamic light scattering spectra, hydrodynamic size and the zeta potential, of NPs. Analysis of (**A**) CeO_2_ (105.7 nm and −32.1 mV), (**B**) AuCeO_2_ (139.2 nm and −29.2 mV) and (**C**) TPP–AuCeO_2_ (216.0 nm and −10.3 mV) performed in water.

AuCeO_2_ and TPP–AuCeO_2_ were synthesized from CeO_2_ according the synthetic route shown in Scheme 1. For this purpose, colloidal CeO_2_ were prepared by hydrolysis of Ce^4+^, following previous reports [20]. Figure S1a shows the characteristic X-ray diffraction peaks of CeO_2_ nanoparticles. The FT-IR spectrum of CeO_2_ was recorded and the vibrations bands appearing at about 500 cm^−1^ and 3300 cm^-1^ were attributed to the vibrations of Ce–O and –OH, respectively (Figure 1). The surface area measured by isothermal N_2_ adsorption was 180 m^2^ g^−1^ and the average particle size of CeO_2_ measured by TEM was 5.2 ± 0.3 nm (Figure S1b) (average of ~100 NPs). The hydrodynamic size and zeta potential of the NPs measured by DLS were 105.7 nm and −32.1 mV, respectively (Figure 2A).

**Figure 3:**
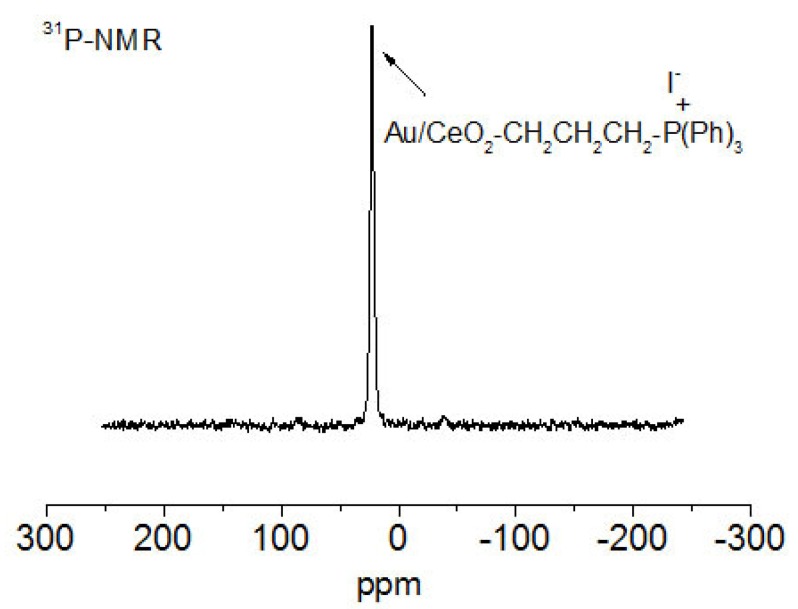
Solid-state ^31^P-NMR spectrum of TPP–AuCeO_2_ confirming the presence of the TPP group in TPP–AuCeO_2_.

Once CeO_2_ had been characterized, AuCeO_2_ was prepared using the deposition-reduction method [20]. The percentage of gold on the AuCeO_2_ solid was 0.1 wt%, based on inductively coupled plasma atomic emission spectroscopy (ICP/AES) measurement. Figure S2 shows the representative DF-STEM images and gold particle size distribution (5.3 ± 3.0 nm) of approximately 100 NPs. The AuCeO_2_ composition was confirmed by DF-STEM analysis coupled to an energy dispersive X-ray (EDX) detector (Figure 1). Figure S3 shows a representative image of AuCeO_2_, its characteristic EDX spectrum and the homogeneous distribution of the elements (Ce, O and Au), as revealed by mapping analyses of each individual component in the sample. It should be noted that due to the large analyzed area of AuCeO_2_ (~500 nm × ~500 nm), it was not possible to observe individual Au NPs as in the case shown in Figure 1. DLS analysis of the AuCeO_2_ sample allowed us to estimate a hydrodynamic size and zeta potential of 139.2 nm and −29.2 mV, respectively (Figure 2B). On the other hand, the FT-IR spectrum of AuCeO_2_ sample showed the presence of –OH groups that are needed for further covalent functionalization with silyl groups (Figure 1). Thus, 3– (iodopropyl)trimethoxysilane was allowed to react with AuCeO_2_ in dry toluene to obtain ω–iodopropyl-functionalized AuCeO_2_ (I–AuCeO_2_). The presence of 3–iodopropyl groups anchored on the AuCeO_2_ surface was shown in the FT-IR spectrum by the presence of new bands at 2980 and 1450 cm^−1^, corresponding to stretching and bending vibrations of –CH_2_, respectively (Figure 1). In addition, new bands around 1150 cm^−1^ could be observed which were attributed to the Si–C and Si–O stretching vibrations. The loading of iodopropylsilyl groups on I–AuCeO_2_ was 4.1 mmol/g, based on the carbon content of this material, as determined by combustion chemical analysis. These iodo groups act as tethers that connect TPP to AuCeO_2_. This reaction was carried out by reacting I–AuCeO_2_ with PPh_3_ to form a strong P–C bond linkage between the positive charged TPP group and AuCeO_2_ (Scheme 1), showing a new approach to achieve TPP functionalization of CeO_2_ and probably other NPs with –OH groups in their structures. The success of this synthesis step was assessed by FT-IR spectroscopy (Figure 1) and ^31^P-NMR (Figure 3). FT-IR spectroscopy revealed that TPP was successfully anchored onto I–AuCeO_2_, as confirmed by the presence of the band at around 1600 and 700 cm^−1^ due to the C=C stretching and C–H bending vibrations caused by the TPP phenyl groups. The ^31^P-NMR spectrum showed a unique signal at 22.96 ppm, corresponding to the TPP units in TPP–AuCeO_2_ [45]. STEM-DF analyses coupled with an EDX detector allowed us to confirm the homogeneous distribution of the different elements present in the TPP–AuCeO_2_ sample (Ce, O, Au and P) (Figure S4). The presence of some residual nonreacted iodide was also detected. As in the case of AuCeO_2_, the analyzed area in the TPP–AuCeO_2_ sample was higher than 500 nm × 500 nm; this fact complicated the observation of the small Au NPs, as in the case shown in Figure S3. DLS measurements allowed us to estimate the hydrodynamic size and zeta potential of the TPP-AuCeO_2_ particles of 216.0 nm and −10.3 mV, respectively (Figure 2C), providing further evidence of the functionalization with TPP moieties. Gold particle size distribution in the TPP–AuCeO_2_ sample was measured by TEM and did not significantly change with respect to AuCeO_2_ samples.

### 3.2. Cell Culture Studies

#### 3.2.1. Cellular Viability and Proliferation

Once the NPs had been successfully obtained, their biocompatibility was evaluated using HeLa cells, in which cell viability and proliferation were assessed. These studies were performed using CeO_2_, AuCeO_2_ and TPP–AuCeO_2_ at two usual concentrations employed in biomedicine (i.e., 10 and 20 µg/mL) [16]. The cytotoxicity of these NPs in HeLa cells was determined by MTT assay at 24, 48 and 72 h of incubation. The cellular viability at 10 µg/mL and 20 µg/mL for each NP showed a slight but nonsignificant decrease in cellular viability and proliferation rate after 72 h, being more pronounced at the higher concentration compared to controls (*p* = 0.085) (untreated cells) (Figure S5). Thus, it can be concluded that the NPs did not produce important cytotoxicity in these cells, and showed suitable biocompatibility for use in biomedical applications.

#### 3.2.2. Cellular Uptake and Internalization of Conjugate by Confocal Microscopy

**Figure 4:**
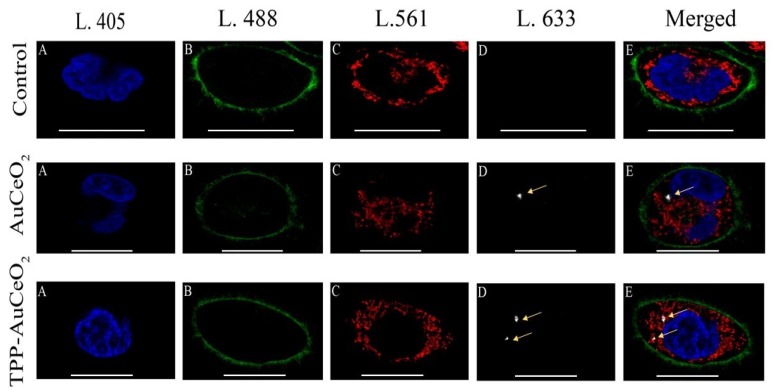
In vivo confocal microscopy images of cellular uptake of AuCeO_2_ and TPP–AuCeO_2_ (20 µg/mL) in HeLa cells after 24h incubation.(**A**) Blue (Hoechst) to label the nucleus; (**B**) Green (CellMask^TM^) to label the cell membrane; (**C**) Red (MitoTracker^TM^) to label the mitochondrial; (**D**) light reflection of NPs at 633 nm (white spots, indicating the location by yellow arrow); and (**E**) colocalization of the cells and the NPs, showing the internalization of these NPs in both treatments (merged). (Scale bar = 20 µm).

Confocal microscopy images confirmed the internalization of AuCeO_2_ and TPP–AuCeO_2_ in HeLa cells. NPs were visualized after irradiating cells with 633 nm laser (white spots in Figure 4D). To determine the cellular localization of the NPs in cells, different cellular organelles, such as the nucleus, cell membrane and mitochondria, were co-stained with blue, green and red fluorescent dyes, respectively (Figure 4A–C). The images revealed the presence of more than one NP per cell in the cellular cytoplasm (for AuCeO_2_) and next to the mitochondria (for TPP–AuCeO_2_) (Figure 4E). Finally, a Z-stack assessment was performed to confirm the internalization and localization of NPs [41,46], in agreement with the previous results (Figure S6).

#### 3.2.3. Mitochondrial Functional Studies after Nanoparticles Treatments

**Figure 5:**
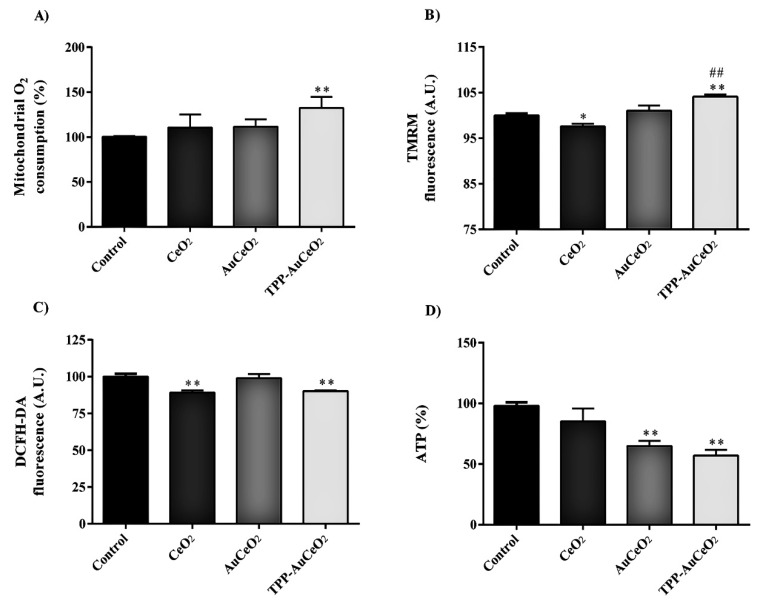
Physiological parameters associated with mitochondrial function. (**A**) Mitochondrial O_2_ consumption of HeLa cells measured in a Clark-type O_2_ electrode, (**B**) ∆Ψm measured with the TMRM probe, (**C**) cellular ROS content measured with the DCFH-DA probe, and (**D**) ATP content measured with the luciferase method after 24 h of treatment with vehicle (control) or CeO_2_, AuCeO_2_, and TPP–AuCeO_2_ at 20 µg/mL. Data are presented as mean ± SEM * *p* < 0.05 versus control group 24 h; ** *p* < 0.01 versus control group 24 h. ^##^ *p* < 0.01 versus CeO_2_.

To determine the effects of CeO_2_, AuCeO_2_ and TPP–AuCeO_2_ at 20 µg/mL on mitochondrial function in HeLa cells after 24 h incubation, we initially measured mitochondrial O_2_ consumption in vitro using a Clark-type O_2_ electrode, and added sodium cyanide to confirm that this consumption had occurred in the mitochondria (Figure 5A). While treatment with CeO_2_ and AuCeO_2_ increased the mitochondrial O_2_ consumption rate slightly (*p* = 0.354 and *p* = 0.196, respectively), TPP–AuCeO_2_ induced a significant increase compared to untreated control cells (*p* < 0.01). This effect was probably related to the predisposition of TPP-functionalized NPs to accumulate near the mitochondria as observed by confocal microscopy.

To further explore the effect of NPs on mitochondrial function, the ∆Ψm of cells was determined using the TMRM probe and measuring the fluorescence in the samples at 690 nm (Figure 5B). A decrease in ∆Ψm in cells treated with CeO_2_ was observed with respect to untreated control cells (*p* < 0.05). However, when Au NPs were supported on CeO_2_, the AuCeO_2_ solid exhibited a slight ∆Ψm increase compared to negative controls. This ∆Ψm increase was higher and statistically significant when cells were treated with TPP–AuCeO_2_ (*p* < 0.01 compared to control cells and *p* < 0.001 compared to CeO_2_-treated cells).

To check if this increase in mitochondrial O_2_ consumption and ∆Ψm signal resulted in changes in ROS production, total cellular ROS content was assessed by measuring fluorescence emission in NP-treated cells at 527 nm (Figure 5C) after addition of DCFH–DA. CeO_2_ and TPP–AuCeO_2_ significantly reduced ROS production (*p* < 0.01 in both cases), but AuCeO_2_ had no significant effect on cellular ROS content, meaning that these NPs do not produce any OS in cells.

The production of ATP in cells after NP treatment (Figure 5D) was estimated using the luciferase method. Our data show that AuCeO_2_ and TPP–AuCeO_2_ were able to significantly reduce ATP production in cells (*p* < 0.01), unlike CeO_2_ that only reduced it slightly (*p* = 0.282).

**Figure 6:**
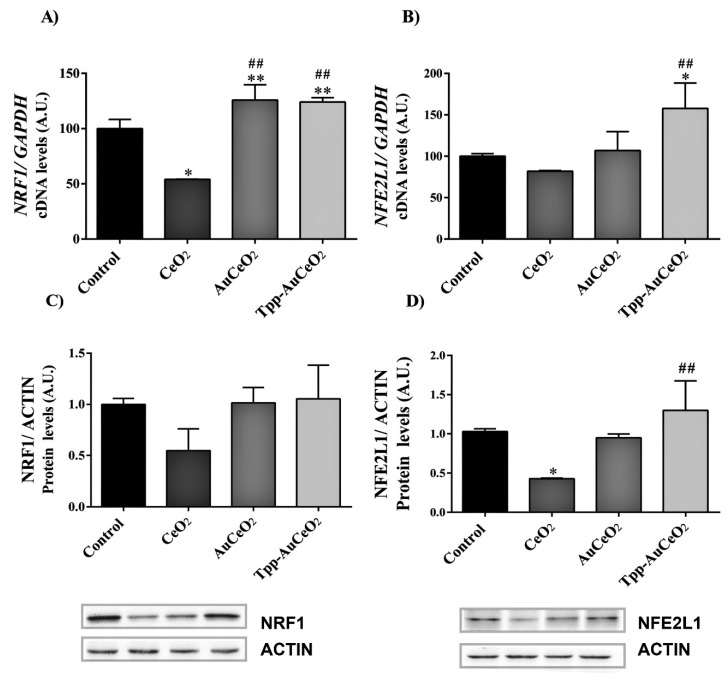
RT-PCR and WB analysis of NRF1 and NFE2L1. Genes expressions of NRF1 (**A**) and NFE2L1 (**B**). Effects on the expression of both genes related to mitochondrial function in HeLa cells after 24h of treatments with CeO_2_, AuCeO_2_ and TPP–AuCeO_2_ (20 µg/mL) and vehicle (control). GAPDH gene expression was used as an endogenous control. Data are presented as mean ± SEM (n = 3). * *p* < 0.05 versus control group; ** *p* < 0.01 versus control group 24 h^. #^ *p* < 0.05 versus CeO_2_. ^#^ *p* < 0.05 versus CeO_2;_^##^ *p* < 0.01 versus CeO_2_. Protein levels of: NRF1 (**C**) (67 kDa), and NFE2L1 (**D**) (85 kDa). ACTIN (45 kDa) was used as an endogenous control. Data are showed as mean ± SEM (n = 3). * *p* < 0.05 versus control.

Additionally, RT-PCR showed a drop in NRF1 and NFE2L1 gene expression after CeO_2_ treatment (Figure 6A,B, respectively), which was only significant for NRF1 (*p* < 0.05). However, the presence of gold supported on CeO_2_ NPs significantly increased the expression of NRF1 (*p* < 0.01). The expression of NFE2L1 also significantly increased after TPP–AuCeO_2_ treatment only (*p* = 0.014), and showed an increasing trend with AuCeO_2_. However, the protein levels of NRF1 and NFE2L1 measured with western blot remained steady in the TPP–AuCeO_2_ group compared to the control group, but fell in the others, being moderate in AuCeO_2_, but considerable and statistically significant in CeO_2_ (*p* < 0.05) (Figure 6C,D, respectively). Finally, the increased of NRFE2L protein was statistically significant regarding the presence of Au, as revealed by the analysis between CeO_2_ and TPP–AuCeO_2_, following a similar tendency to that observed in the expression of this gene (Figure 6D).

## 4. Discussion

As previously noted, this manuscript aims to report the synthesis of CeO_2_, AuCeO_2_ and TPP–AuCeO_2_, as well as the evaluation of the effects of these NPs on mitochondrial function. Initially, CeO_2_ and AuCeO_2_ nanoparticles were obtained following a methodology previously described by our group [20]. In order to covalently functionalize AuCeO_2_ with TPP units, a new, two-step synthetic methodology was performed. This methodology is based on the functionalization of –OH groups present in the surface of the AuCeO_2_ solid through a silylation reaction to attach the iodoalkyl chain by strong covalent Ce–O–Si bonds, and subsequently perform a nucleophilic substitution reaction of iodide with PPh_3_. This methodology can be used to functionalize other similar NPs with the TPP group as long as they have –OH groups in their surface. Thus, the –OH groups of AuCeO_2_ were used to attach iodopropyl groups, thereby resulting in I–AuCeO_2_. Subsequently, the iodo group present in the I–AuCeO_2_ sample was reacted with PPh_3_, resulting with the formation of TPP–AuCeO_2_. The characterization data of all the NPs confirmed that Au deposition and the functionalization procedure did not significantly alter the size of CeO_2_, while zeta potential was less negative in the presence of Au or positive when NPs were functionalized with the TTP group.

Once NPs had been synthesized and characterized, the internalization of AuCeO_2_ and TPP–AuCeO_2_ was assessed by confocal microscopy. Since the internalization of both CeO_2_ and AuCeO_2_ in cells had been already confirmed in a previous study [20], we wanted to assess the ability of TPP–AuCeO_2_ to internalize and the effect caused by the presence of the TPP group when compared with unfunctionalized AuCeO_2_. Confocal microscopy z-stack analysis revealed that AuCeO_2_ and TPP–AuCeO_2_ were located mainly in the cytoplasm and next to the mitochondria, respectively. Furthermore, a biocompatibility study performed with all synthesized NPs confirmed that they did not affect viability and proliferation after 72 h of incubation in HeLa cells. The effects of these three types of NPs on mitochondrial function were subsequently assessed in HeLa cells. Treatment with these NPs resulted in an increase in mitochondrial O_2_ consumption, being only statistically significant with the presence of the TPP counterpart. This effect could be valuable in the treatment pathological conditions that require an increase of mitochondrial activity, such as diabetes, cancer, Alzheimer’s disease or obesity [47,48,49]. To further investigate the mitochondrial effect caused by these NPs, the cellular ∆Ψm was also measured. The behavior of NPs against ∆Ψm was completely different depending on the presence of Au in NPs; it decreased significantly with CeO_2_ and tended to increase with AuCeO_2_, being significantly higher with TPP–AuCeO_2_. These results suggest a change in mitochondrial function of cells induced by the presence of Au in CeO_2_ NPs that was increased by the proximity of these NPs to mitochondria after their TPP-functionalization. This data indicates that the presence of Au in CeO_2_ can notably alter notably mitochondrial function in cells. Another parameter related to mitochondria in eukaryotic cells is cellular ATP production, since it is mainly produced in the mitochondria. ATP production was found to be reduced in all NP treatment groups, but was only statistically significant for AuCeO_2_ and TPP–AuCeO_2_. These observations demonstrate that Au enhances ATP reduction when NPs accumulate near the mitochondria. To further explore if these effects on mitochondrial function affected cellular OS, ROS content was measured in cells after incubation with NPs. None of the NPs caused an increase in ROS content. In fact, the ROS content was significantly reduced by the presence of CeO_2_ and TPP–AuCeO_2_, and showed a trend toward reduction when incubated with AuCeO_2_. Therefore, these NPs did not enhance OS in cells despite altering the mitochondrial function. We suggest that the presence of Au in AuCeO_2_ and TPP–AuCeO_2_ NPs induced a change in the cellular mitochondrial function, thereby modulating its activity. In addition, TPP–AuCeO_2_ exhibited a similar tendency to AuCeO_2_ which was considerably higher and more noticeable than when only TPP moieties were present in NPs. This is the first time that this kind of behavior has been reported, which raises the possibility of modulating the mitochondrial action of the antioxidant CeO_2_ by Au. Thus, this work opens new avenues to synthesize personalized antioxidants based on CeO_2_ to treat diseases after taking into account mitochondrial needs, thereby enhancing the biomedical applicability of these NPs.

Our analysis was completed by studying the expression of NRF1 and NFE2L1 genes whose cellular expression was completely changed by the presence of Au in the NPs. The expression of both genes was reduced when cells were treated with CeO_2_, but increased when incubated with AuCeO_2_ and TPP–AuCeO_2_. NRF1 is an important gene for mitochondrial function, since it promotes the expression of genes related to mitochondrial respiration, mitochondrial biogenesis and mitochondrial DNA transcription and regulation [45,46,50]. Changes in NRF1 expression as a result of NP treatment may be associated with previously observed changes in cellular respiration and ATP production. The treated cells showed changes in NRF1 gene expression. Our results reveal that the presence of Au in CeO_2_ NPs increases the expression of this gene that is depleted in presence of CeO_2_, probably causing the alterations observed in ∆Ψm and ATP production. On the other hand, NFE2L1 acts as a transcription factor by binding to antioxidant response element sequences, thus regulating the expression of several genes involved in antioxidant defenses [51,52]. Our data confirm again that the presence of Au alters mitochondrial function, and its presence increases the expression of NFE2L1, which was reduced by CeO_2_, and therefore the expression of genes involved in the production of endogenic antioxidants. The fact that TPP–AuCeO_2_ can significantly enhance the expression of NRF1 and NFE2L1 highlights the potential of these NPs as modulators of mitochondrial function and OS in cells. In addition to RT-PCR experiments, the protein content of NRF1 and NFE2L1 in cells after NP treatment was measured by western blot. Both analyses showed a tendency of CeO_2_ to decrease the content of both proteins, being statistically significant in NFE2L1, while both proteins were increased in the presence of Au, with a greater increase when functionalized with TPP. Thus, the protein content seems to be aligned with the expression of genes, corroborating the outcomes.

Our findings show that the mitochondrial action mechanism of CeO_2_ in cells was modified by Au, and was more noticeable when it was targeted to mitochondria through functionalization with the TPP group. Therefore, although all these NPs have been proposed as antioxidants, their effects on mitochondrial function are completely different. Thus, these NPs can be used as personalized antioxidants agents to treat different diseases depending on their effect on the mitochondrial function. It should be mentioned that several pathologies require different antioxidants based on their effect on mitochondrial function. Nevertheless, further studies are required to gain a better understanding about the cellular mechanism and pathways affected by these NPs, especially those related with mitochondrial effects due to the TPP–AuCeO_2_ (Figure 5).

## 5. Conclusions

In summary, we have successfully established a new methodology for the functionalization of ceria nanoparticles with TPP groups. In addition, the effects of CeO_2_, AuCeO_2_ and TPP–AuCeO_2_ on the cellular mitochondrial function have been successfully assessed. For the first time, we have demonstrated that the presence of Au in CeO_2_ nanoparticles can modulate the effects on the mitochondrial function caused by CeO_2_ in cells. This effect can be enhanced by increasing the mitochondria-targeting ability of AuCeO_2_ through functionalization with TPP groups. This modulation of mitochondrial function may be used to enhance the therapeutic utility of these NPs for various biomedical applications.

## Supplementary Materials

The following are available online at https://www.mdpi.com/2079-4991/10/4/744/s1, Figure S1. Powder XRD (performed in a Shimadzu XRD-7000 diffractometer using Cu Kα at a scanning speed of 1° per min in the 10 – 80° 2θ range (Shimadzu Europa GmbH, Duisburg, Germany) and representative TEM images of CeO_2_; Figure S2. TEM image of AuCeO_2_ with size distribution of Au NPs; Figure S3. TEM of AuCeO_2_ sample with its EDX spectrum and mapping of Au, Ce, O, Cu and C; Figure S4. TEM image of TPP–AuCeO_2_ with its EDX spectrum and mapping of Au, Ce, O, Cu, C, Si, P, and I; Figure S5. Effects of CeO_2_, AuCeO_2_ and TPP–AuCeO_2_ on cellular proliferation and viability in cells after 24, 48 and 72 h; Figure S6. Z-stack images of HeLa Cells incubated without NPs and with AuCeO_2_ and TPP–AuCeO_2_.
