# Supplementary material for: Alteration of the Mitochondrial Effects of Ceria Nanoparticles by Gold: An Approach for the Mitochondrial Modulation of Cells Based on Nanomedicine
Source: Nanomaterials (Basel). 2020 Apr 13;10(4):744. doi: 10.3390/nano10040744 (PMC7221686; doi:10.3390/nano10040744)
Supplement: Supplementary file 1 [file nanomaterials-10-00744-s001.pdf]

nanomaterials

MDPI

Suplemmentary material

Alteration of the Mitochondrial Effects of Ceria Nanoparticles by Gold: An Approach for the Mitochondrial Modulation of Cells Based on Nanomedicine

Patricia Gutiérrez-Carcedo [1,2], Sergio Navalón [3], Rafael Simó [2], Xavier Setoain [4], Carolina Aparicio-Gómez [1], Ibane Abasolo [5], Victor Manuel Victor [6,7], Hermenegildo García [3,*] and José Raúl Herance [1,*]

[1] Medical Molecular Imaging Research Group, Vall d'Hebron Research Institute, CIBBIM- Nanomedicine, Universitat Autònoma de Barcelona (UAB) and Biomedical Imaging Group, Biomedical Research Networking Center in Bioengineering, Biomaterials and Nanomedicine (CIBER-BBN), Barcelona 08035, Spain; biotecpat@gmail.com (P.G.-C.); carolina.aparicio@vhir.org (C.A.-G.)

[2] Diabetes and Metabolism Research Unit, Vall d'Hebron Research Institute, Department of Endocrinology, Vall d'Hebron Research Institute, UAB, Biomedical Research Center in Diabetes Network and Associated Metabolic Diseases (CIBERDEM), Barcelona 08035, Spain; rafael.simo@vhir.org

[3] Deparment of Chemistry and Instiute of Chemical Technology (CSIC-UPV), Universitat Politècnica de València, Valencia 46022, Spain; sernaol@doctor.upv.es

[4] Hospital Clinic, Biophysics and Bioengineering Unit, Biomedicine Department, School of Medicine, University of Barcelona, and CIBER-BBN, Barcelona 08036, Spain; setoain@clinic.cat

[5] Functional Validation & Preclinical Research (FVPR), Group of Drug Delivery & Targeting, CIBBIM-Nanomedicine, Vall d'Hebron Research Institute, UAB, CIBBER-BBN, Barcelona 08035, Spain; ibane.abasolo@vhir.org

[6] Service of Endocrinology and Nutrition. University Hospital Doctor Peset, FISABIO, Valencia 46017; victor.victor@uv.es

[7] CIBERehd, Department of Physiology, University of Valencia, Valencia 46010, Spain

* Correspondence:hgarcia@qim.upv.es (H.G.); raul.herance@vhir.org (J.R.H.); Tel.: +34-96-387-7807 (H.G.); Tel.: +34-93-489-3000 (ext: 4946) (J.R.H.)

Received: 3 March 2020; Accepted: 7 April 2020; Published: date

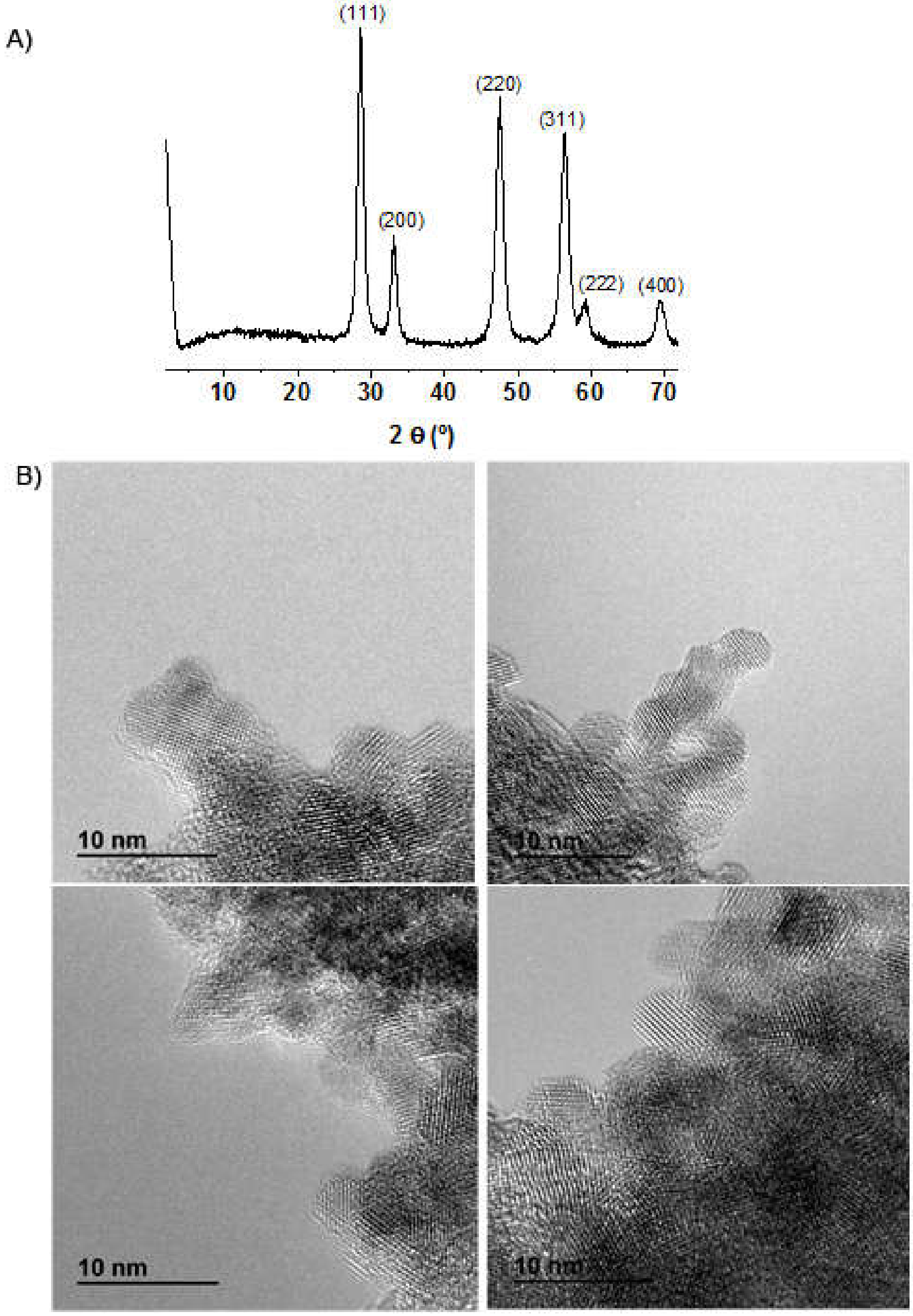

Figure S1. (**A**) Powder XRD and (**B**) representative TEM images of CeO_2 nanoparticles.

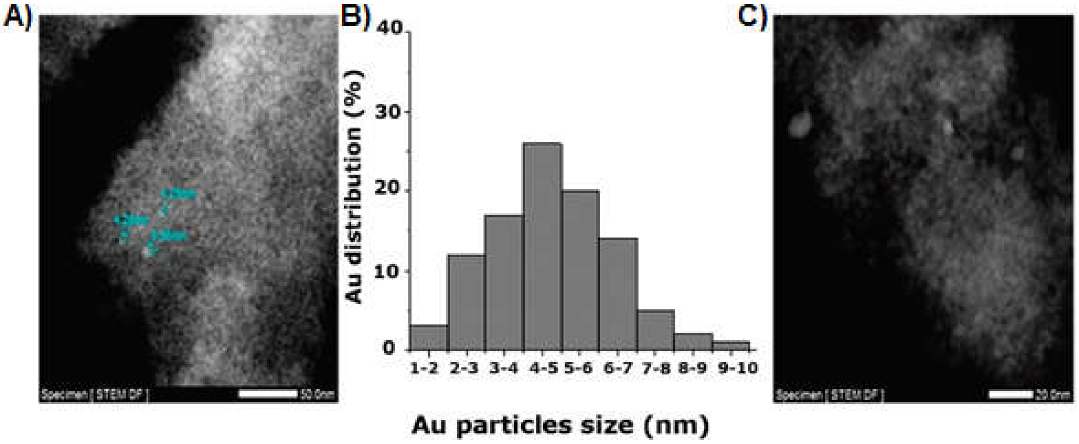

Figure S2. (**A**), (**C**) TEM image corresponding to $AuCeO_2$ sample; (**A**–**C**) The particle size distribution of Au NPs, (Scale bar = 50 nm, left), (Scale bar = 20 nm, right)

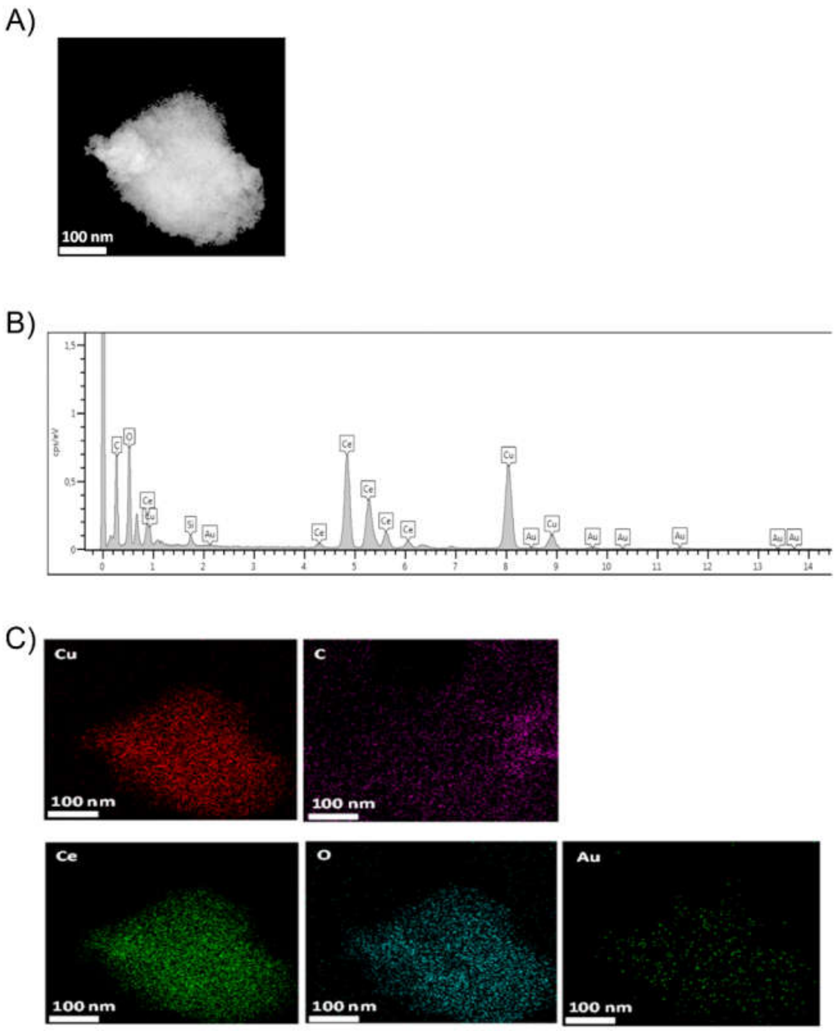

Figure S3. TEM image corresponding to: (**A**) $AuCeO_2$, (**B**) EDX spectrum, and (**C**) mapping of the different elements present in panel A.

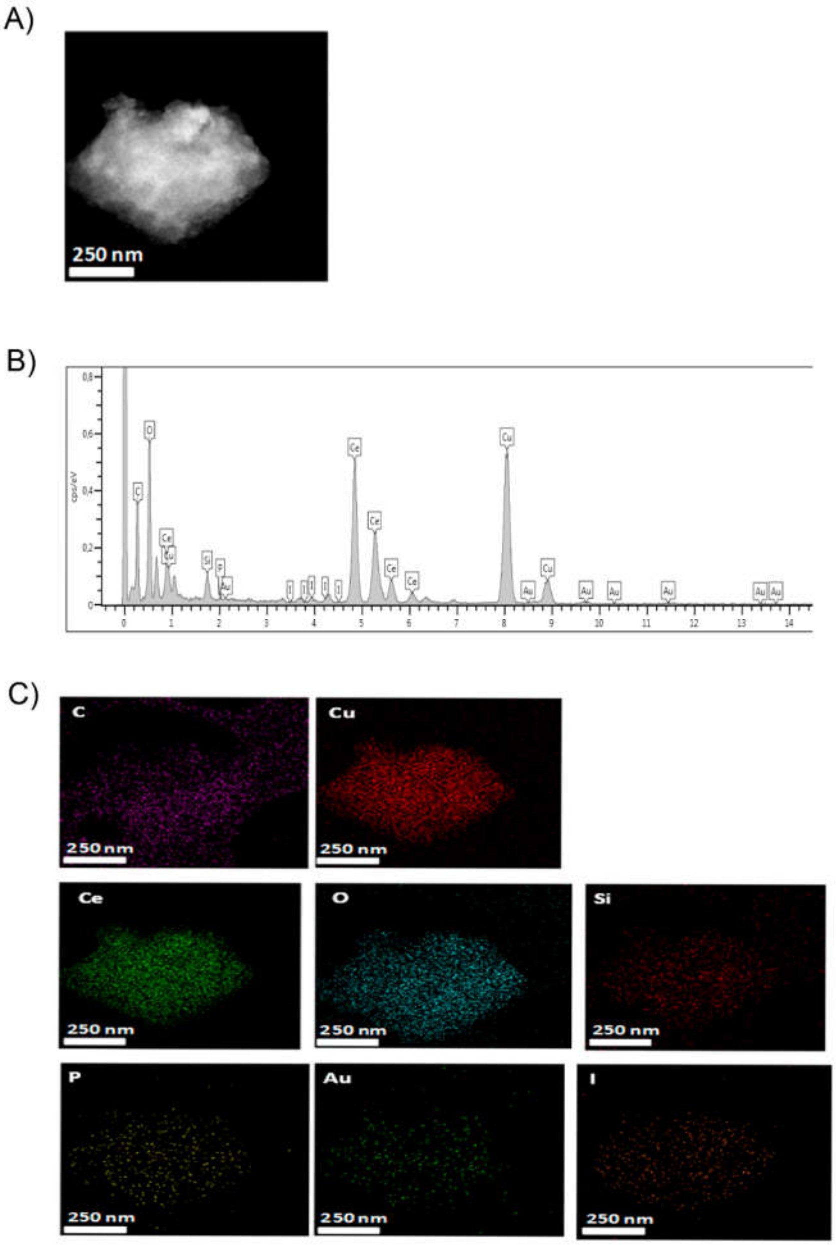

Figure S4. TEM image corresponding to: (**A**) TPP–AuCeO_2, (**B**) EDX spectrum, and (**C**) mapping of the different elements present in panel A.

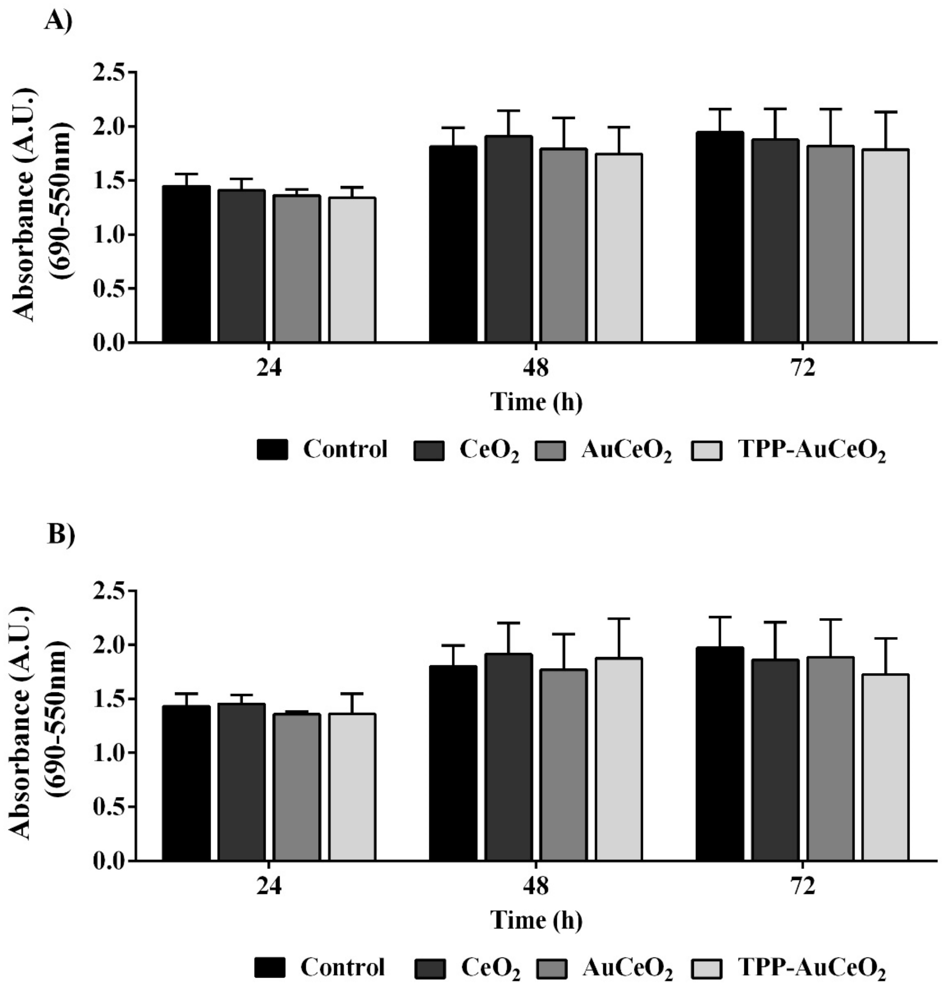

Figure S5. Determination of cellular viability and proliferation. Effect of TPP–AuCeO2, AuCeO2 and CeO2 on cellular proliferation and viability in HeLa cells assessed by MTT assay after 24, 48 and 72 h incubation. (**A**) 10 μg/mL, (**B**) 20 μg/mL.

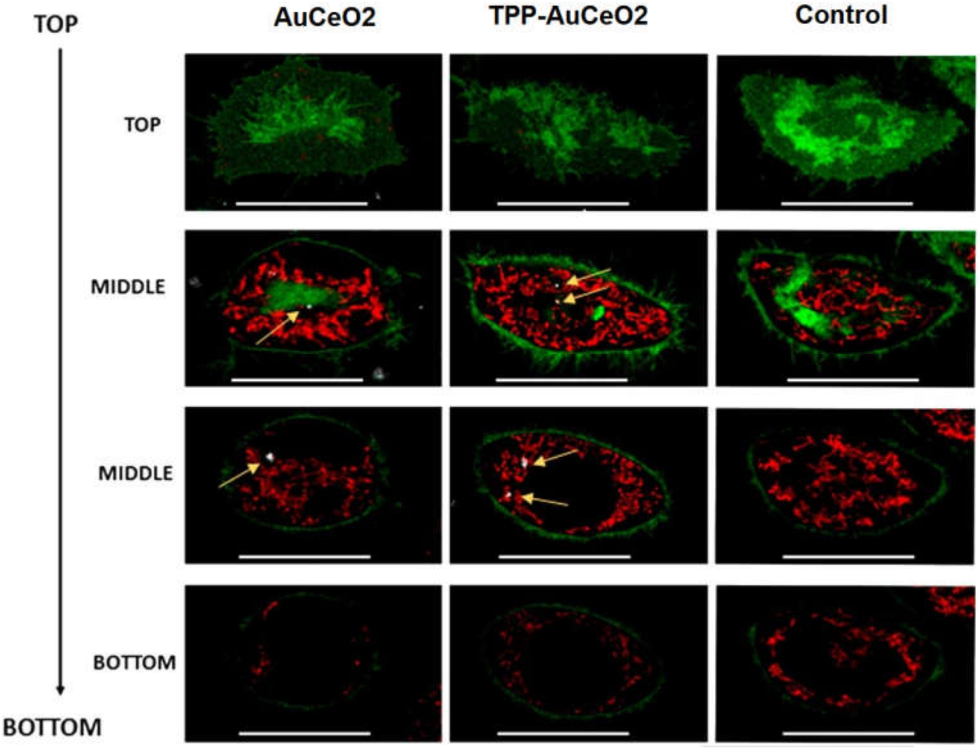

Figure S6. Z-axis scanning images of $AuCeO_2$, TPP–$AuCeO_2$ (20 μg/mL) and vehicle in HeLa cells by laser confocal microscopy obtained merging the green (CellMask™) to label the cellular membrane and red (MitoTracker™) to label the mitochondrial. Fluorescence were obtained exciting at 488 nm (exciting CellMask™, green) and 561nm (exciting MitoTracker™, red) and the emission was collected from 425 to 603 nm in separate channels. The white spots indicate the location of NPs after irradiating at 633 nm (yellow arrows). (Scale bar = 20 μm).

© 2020 by the authors. Submitted for possible open access publication under the terms and conditions of the Creative Commons Attribution (CC BY) license (http://creativecommons.org/licenses/by/4.0/).
